# Association between Active *H. pylori* Infection and Iron Deficiency Assessed by Serum Hepcidin Levels in School-Age Children

**DOI:** 10.3390/nu11092141

**Published:** 2019-09-07

**Authors:** Eugenia Mendoza, Ximena Duque, Jordán I. Hernández Franco, Elba Reyes Maldonado, Segundo Morán, Gloria Martínez, Aarón Salinas Rodríguez, Homero Martínez

**Affiliations:** 1 Infectious Diseases Research Unit, Gastroenterology Research Laboratory, Research Unit in Epidemiology and Health Services, Mexican Institute of Social Security, 06725 Mexico City, Mexico (E.M.) (S.M.) (G.M.); 2 Morphology Department, National School of Biological Sciences, Instituto Politécnico Nacional, 01135 Mexico City, Mexico (J.I.H.F.) (E.R.M.); 3 Centro de Investigación en Evaluación y Encuestas, INSP, 62100 Cuernavaca, Mor, Mexico; 4 Global Technical Services-NTEAM, Nutrition International, Ottawa, ON K2P 2K3, Canada; 5 Dirección de Investigación, Hospital Infantil de México “Federico Gómez”, 06720 Mexico City, Mexico

**Keywords:** serum hepcidin, *H. pylori* infection, iron deficiency, school-age children

## Abstract

Hepcidin regulates iron metabolism. Its synthesis increases in infection and decreases in iron deficiency. The aim of this study was to evaluate the relationship between *H. pylori* infection and iron deficiency by levels of hepcidin in children. A total of 350 school-age children participated in this cross-sectional study. Determinations of serum ferritin, hemoglobin, hepcidin, C-reactive protein, and α-1-acid-glycoprotein were done. Active *H. pylori* infection was performed with a ^13^C-urea breath test. In schoolchildren without *H. pylori* infection, hepcidin was lower in those with iron deficiency compared to children with normal iron status (5.5 ng/mL vs. 8.2 ng/mL, *p* = 0.017); while in schoolchildren with *H. pylori* infection the levels of hepcidin tended to be higher, regardless of the iron nutritional status. Using multivariate analysis, the association between *H. pylori* infection and iron deficiency was different by hepcidin levels. The association between *H. pylori* and iron deficiency was not significant for lower values of hepcidin (Odds Ratio = 0.17; 95% Confidence Interval [CI] 0.02–1.44), while the same association was significant for higher values of hepcidin (OR = 2.84; CI 95% 1.32–6.09). This joint effect is reflected in the adjusted probabilities for iron deficiency: Individuals with *H. pylori* infection and higher levels of hepcidin had a probability of 0.24 (CI 95% 0.14–0.34) for iron deficiency, and this probability was 0.24 (CI 95% 0.14–0.33) in children without *H. pylori* infection and lower levels of hepcidin. In children with *H. pylori* infection and iron deficiency, the hepcidin synthesis is upregulated. The stimulus to the synthesis of hepcidin due to *H. pylori* infection is greater than the iron deficiency stimulus.

## 1. Introduction

Hepcidin is a hormone formed by 25 amino acids, produced mainly by hepatocytes. It has been identified as the main regulator of iron absorption in the body [1,2]. Through the hepcidin/ferroportin-1 system, it irreversibly joins with the ferroportin-1 of enterocytes and macrophages, inducing its internalization and later lysosomal degradation, which reduces iron efflux. Hepcidin is also related to reduction of divalent metal transporter 1 (DTM1) transcription, which is how it regulates iron absorption in the diet, as well as storage and release of iron in or from deposits [2,3]. Hepcidin production increases when there are high concentrations of iron, and its synthesis is inhibited by hypoxia, erythropoiesis, iron deficiency, and iron deficiency anemia [3,4]. Hepcidin is also a type II acute phase peptide. Its production is increased by the stimulus of IL-6 and IL-1 beta during infectious inflammatory processes. 

*Helicobacter pylori* is a Gram-negative bacteria that colonizes the gastric mucous of approximately 50% of the world population [5]. Infection with *H. pylori* has been associated with the development of gastrointestinal diseases such as active chronic gastritis, peptic ulcers, and gastric cancer [5,6]. It has also been associated with extra-gastric manifestations, such as thrombocytopenic purpura, reduction in growth velocity, and iron deficiency and/or anemia [7,8]. Some studies have reported that by eradicating *H. pylori* bacteria, indicators of iron nutritional status return to normal values without the need for iron supplementation [9]. The Consensus of Maastricht V/Florence recommends eradication treatment in patients with iron deficiency anemia that cannot be explained by other causes and is associated with gastritis in the presence of *H. pylori* because there is evidence in children and adults of an association between *H. pylori* and iron deficiency anemia [10].

The mechanism or mechanisms through which *H. pylori* infection may cause iron deficiency and/or anemia are not fully understood yet. Potential mechanisms include: increase in intragastric pH; reduced concentration of ascorbic acid in gastric juices, which affects iron absorption from the diet; chronic bleeding produced by the development of micro-erosions in gastric mucous; production of lactoferrins by neutrophils; and capture of iron by the bacteria [11]. Another mechanism could be the increase in the synthesis of hepcidin, a central regulator of iron metabolism that blocks iron absorption in the small intestine [12,13,14]. The increase in the production of cytokines IL-6 and IL-1beta, mediated mainly by stimulation of the A component of the lipopolysaccharide of *H. pylori*, may stimulate the production of hepcidin in hepatic cells. Consequently, iron absorption and mobilization of deposits from the liver and of macrophages is reduced, causing a reduction in the metal available at the serum level. This may lead to the development of iron deficiency and anemia, as has been observed in other infectious processes [15,16,17,18]. 

In settings where children frequently have infectious diseases and may also present iron deficiency, sorting out the role of infection or chronic inflammation in the children’s iron nutrition status is a challenge because the concentration of various indicators of iron nutritional status is affected by these processes. For example, low serum ferritin is commonly used as an indicator of low body iron stores. However, ferritin is also an acute phase reactive protein, so it increases during infectious processes. An increase in ferritin production in the presence of infection reduces the availability of iron to microorganisms, contributing to nutritional immunity [19]. Therefore, when evaluating iron nutritional status using ferritin concentrations, it is recommended to control for the potential confounding influence of inflammation by excluding from the analysis individuals with high levels of acute phase proteins such as C-reactive protein (CRP) and α-1-acid glycoprotein (AGP) [20]. However, the concern of using this strategy is that infections, inflammatory process, and iron deficiency occur simultaneously in developing countries.

The objective of this study was to evaluate the possible relationship between *H. pylori* infection and iron deficiency by levels of hepcidin in school-age children, controlling for positive acute-phase proteins, including CRP and AGP. 

## 2. Materials and Methods

### 2.1. Study Population

Three hundred and fifty schoolchildren participated in this study. They ranged in age from 6 to 14 years old and were part of a parental, longitudinal study looking at the effect of *H. pylori* infection on growth speed in schoolchildren [21]. These children attended three public boarding schools in Mexico City, where students stay five days a week and return home on weekends and holidays. These boarding schools are run by the Secretary of Public Education and cater to children of families with limited resources. 

For this study, there was a random selection of participants. Inclusion criteria included making sure that the serum samples had not undergone freezing and thawing cycles, as samples were separated in various aliquots before freezing, and that the serum sample would be sufficient for biochemical determinations. 

### 2.2. Determination of Active H. pylori Infection and Hematological Parameters

Information on *H. pylori* infection and hematological parameters was obtained from the parent study. Active *H. pylori* infection was determined by breath test with ^13^C-urea, this test consisted of collecting two samples of expired air. The basal sample was obtained 10 min after a child had ingested a beverage containing 2 g of citric acid (Citra-LP, San Miguel Proyectos Agropecuarios S.P.R., Hidalgo, Mexico) to delay gastric emptying. Immediately afterward, child was given 50 mg of ^13^C-labeled urea dissolved in 150 mL of water, and the final sample was collected after 30 min. The ^13^CO_2_:^12^CO_2_ ratio was calculated for the samples before and after the ingestion of 13C urea. A change of ≥5 parts/1000 was considered positive, as previously published [21]. Children with *H. pylori* did not receive eradication treatment for this condition. At present, there is no indication to treat asymptomatic individuals and without other risk backgrounds that show an infection with *H. pylori* [10]. 

We also used data on hemoglobin and serum ferritin concentration. The blood samples were drawn under 8-h fasting conditions, on the same day in which the breath sample was collected for detecting active *H. pylori* infection. Venous blood was collected in tubes with Ethylenediamine tetraacetic acid (EDTA), for hemoglobin determination, and in vacutainer tubes for serum; samples were centrifuged at 4000 rpm, and serum aliquots were stored at −80 °C in appropriate air-tight tubes since their initial collection until they were used for biochemical determinations. Samples were never subject to freezing and thawing cycles before being used for this study. Determinations included C-Reactive protein (CRP), α-1 glycoprotein (AGP), and serum hepcidin.

Hemoglobin (Hb) concentration was determined by automated analyses using cyanomethemoglobin method (Beckman Coulter T-540, Brea, CA, USA). Serum ferritin was determined by radioimmunoassay (FER-IRON II, RAMCO LABORATORIES, Stafford, TX, USA). Following recommendations from the World Health Organization, iron deficiency was defined as a ferritin ˂ 15 µg/L and anemia was defined as Hb ˂ 11.5 g/dL in children less than 12 years and Hb < 12.0 g/dL in children aged 12 years or older [22,23]; hemoglobin concentration was adjusted by altitude using the Ruiz-Argüelles equation: %HB = (93.3197) (10 ^0.0000251*altitude in meters^) [24].

Determinations of CRP and AGP in serum were performed by ELISA (GWB-F43C70, GenWay Biotech. Inc. San Diego, CA, USA, and RAP001 and Biovendor Research and Diagnostic Products, Asheville, NC, USA respectively). Concentrations ˂ 5 mg/L were considered normal for CRP and < 1 g/L for AGP [25].

Hepcidin was measured in serum by competition ELISA, using the hepcidin-25 (human) enzyme immunoassay kit (S-1337; Bachem, San Carlos, CA, USA) with detection range 0–25 ng/mL, according to the manufacturer’s protocol. Standards were run in duplicate and single sample determination was made. Samples giving readings outside the linear region of the curve were diluted 1:4 in peptide-cleared human sera provided with the kit. Hepcidin-25 levels were calculated from a calibration curve with a linear measuring range near the IC50 of the curve 1.5 ng/mL (0.35–1.72). It has been observed that hepcidin maintains its structure in serum or plasma at room temperature for at least one day and remains stable over long term at −80 °C. In a study that analyzed samples kept frozen for 9–11 years at −80 °C compared with fresh samples matched by age and gender, there were no statically significant differences in hepcidin concentration, which suggests that this hormone is stable for long periods of time [26]. The presence of four disulfide bridges in its structure may be related to its stability [27]. 

At present, there are no cut-off points to define normal, high, or low hepcidin levels, therefore the concentration is described with the median and interquartile range.

### 2.3. Anthropometric Measurements

According to the technique suggested by Lohman and others [28], height and weight were recorded for children without shoes and wearing light clothes, standing straight, with their weight uniformly distributed on both feet and their arms hanging freely on the sides. A digital scale (model 881; Seca, Hamburg, Germany) with a reading precision of 50 g for weights up to 50 kg and of 100 g for greater weights was used. Accuracy of the scale was maintained by using calibration weights. Height measurements were reported in centimeters, approximating the reading to the nearest 0.1 cm. An Easy Glide Bearing wall stadiometer (Perspective Enterprises, Portage, MI, USA) with a reading precision of 1 mm was used. Before starting each work session, the stature meter was checked for calibration using a standard 60-cm stick. 

To evaluate the nutritional status by anthropometric indicators, AnthroPlus software was used. The indicator of height to age in *Z* score was obtained and a categorical variable was generated with the following categories: ‘normal’ includes observations with *z* scores ≥ −1 SD, risk of stunting or stunting includes observations with *z* scores less than −1 SD. Body mass index (BMI) to age was obtained, and a categorical variable was generated as follows: ‘normal’ includes observations with a *z* score < 1 SD but > −2 SD; overweight/obesity includes observations with a *z* score ≥ 1 SD [29].

### 2.4. Ethical Considerations

To participate in the present study, parents or tutors signed an informed consent authorizing participation of their children. Children seven years old or more gave their assent in writing. In the main study all children who presented iron deficiency received therapeutic iron supplementation in the form of 30 mg/day of elemental iron for 3 months. The use of serum samples and data from the main study for the specific aim of this analysis was approved by the Research and Ethics Committee of the Mexican Institute of Social Security (Register number R-2014-785-023). 

### 2.5. Statistical Analysis

Characteristic of the study population by iron status are described using media ± SD, median and percentiles 25 and 75, and percentages taking account the variables distribution. Hepcidin, CRP, and AGP measurements do not have a normal distribution. Age, BMI, height for age (*Z* scores), and hemoglobin were compared by iron status using the Student *t*-test. Hepcidin, CRP, and AGP concentrations were compared by iron status using the Wilcoxon test for independent samples. Categorical variables were compared by the Pearson *χ*^2^ test. Hepcidin levels were compared by iron nutritional status, anthropometric indicators, *H. pylori* infection, and inflammatory markers; in these comparisons, the Wilcoxon test for independent samples was used. 

The association between *H. pylori* infection and iron deficiency by serum hepcidin levels was assessed at multivariate analysis using a logistic regression model. The first stage of this analysis consisted of finding the adequate functional form to include hepcidin levels in the interaction term. Results from the simple analysis between *H. pylori* infection and hepcidin levels and its association with iron deficiency, as well as the literature reviewed, suggest a potential interaction between these two variables. However, given the limited knowledge about the usual values of hepcidin in the general population, even more so in children, it was necessary to carry out an exploratory analysis to look for the most adequate function to introduce this variable in the logistic analysis. Initially, we considered introducing the hepcidin variable as a continuous one, but hepcidin evidence from different studies on the regulatory role of hepcidin on iron metabolism leads us to consider the possibility that at low values of hepcidin there may be iron deficiency, while at high levels there may be an infectious or inflammatory process in place, which leads to a reduction in iron absorption. For these reasons, and given the lack of reference cut-offs, hepcidin’s values were categorized in quartiles. Next, a dichotomous variable was created, with one value assigned to the lowest quartile and the other to the second to fourth quartiles. This categorization allowed us to clearly show the interaction between the two terms in association with iron deficiency. Furthermore, a multivariate model was built, adjusting for other variables. To assess the fit of model Pearson *χ*^2^, goodness-of-fit was used. Marginal predicted probabilities to iron deficiency from the logistic model were obtained. Statistical analyses were performed using the Stata version 12 software (Stata Corporation, Collage Station, TX, USA). 

## 3. Results

Our study population was composed of 350 schoolchildren; 55.1% (*n* = 193) were female; 56 (16.0%) had low ferritin levels, a variable that identifies low iron storages and is commonly used as proxy variable of iron deficiency. Seventy-seven (22%) children had active *H. pylori* infection (^13^C-Urea breath tests); 24% (*n* = 84) were stunted or at risk of stunting, and 34% (*n* = 119) were overweight or obese. A higher percentage of children with iron deficiency also had stunting risk or stunting compared with those with normal iron status (42.9% vs. 20.4%, *p* = 0.000). The percentage of children with *H. pylori* infection also was higher between those with iron deficiency compared to children with normal iron status (35.7% vs. 19.4, *p* = 0.007). The children with iron deficiency were younger than those with no deficiency (9.1 ± 1.8 vs. 9.8 ± 1.8 years, *p* = 0.009). The hemoglobin concentration was similar between children with and without iron deficiency; none of the children had anemia. Hepcidin levels were slightly lower in children with iron deficiency (6.9 ng/mL vs. 8.6 ng/mL), but not statistically significantly different (*p* = 0.261). A larger percentage of children had normal (88.8%) versus high (11.2%) CRP levels, independent of iron status; the same was observed with respect to AGP, with 88.3% of children having normal versus 11.7% showing high values, independent of iron status (see Table 1).

Children with *H. pylori* infection had higher serum hepcidin levels than those without *H. pylori* infection, but there was no significant difference in hepcidin levels between children with *H. pylori* infection by iron status (10.5 and 10.4 ng/mL, *p* = 0.693). In children without the *H. pylori* infection, hepcidin levels were lower in those with iron deficiency compared with the group with normal iron status (5.5 and 8.2 ng/mL, *p* = 0.017). In the children with iron deficiency, hepcidin levels tended to be lower in those without *H. pylori* infection compared to those with the infection (5.5 and 10.5 ng/mL, *p* = 0.094) (Table 2). 

Hepcidin levels were higher in children with high CRP than in children with normal CRP but only in the group with normal iron status (15.0 vs. 8.4 ng/mL, *p* = 0.016), while hepcidin levels were higher in children with high α-1 glycoprotein, but only in the group with iron deficiency (10.5 vs. 6.8, *p* = 0.044) (Table 2).

Iron status, hepcidin levels, and inflammation markers by *H. pylori* infection are presented in Table 3. Hemoglobin concentration was lower in children with *H. pylori* infection (14.0 ± 0.8 g/dL vs. 14.2 ± 0.8 g/dL, *p* = 0.017). Ferritin level were not different by *H. pylori* infection, but the percentage of children with iron deficiency was higher in those with *H. pylori* infection (26.0% vs. 13.2%, *p* = 0.007). Hepcidin levels were higher in children with *H. pylori* infection compared to children without the infection (median 10.4 ng/mL vs. 7.5 ng/mL, *p* = 0.005). The higher percentage of children with *H. pylori* infection had high levels of α-1 glycoprotein compared with children without this infection (24.7% vs. 8.1%, *p* = 0.000). 

**Figure 1:**
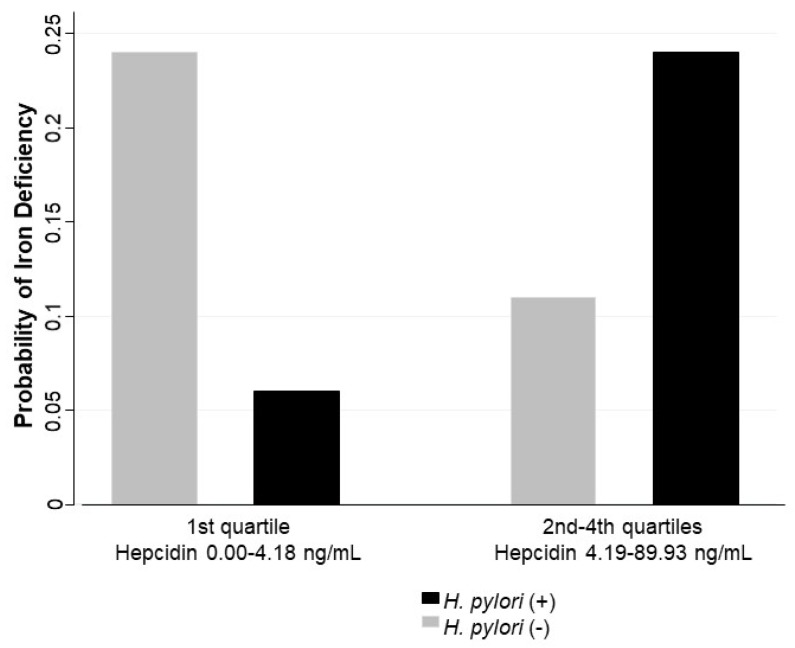
Predicted probabilities to iron deficiency by *H. pylori* infection and hepcidin categories, multivariate model Table 4.

In the multivariate analysis adjusted by age, stunting or risk to stunting versus normal height for age, high versus normal CRP, high versus normal α1-glycoprotein, sex, and overweight/obesity versus normal body mass index, an interaction by hepcidin levels was identified in the association between *H. pylori* infection and iron deficiency. Hepcidin levels were included in the model using a dichotomous categorization: first quartile (values between 0.00 and 4.18 ng/mL) and second to fourth quartiles (values between 4.19 to 89.93 ng/mL). The association between *H. pylori* and iron deficiency was not significant for lower values of hepcidin (OR = 0.17; 95% CI 0.02–1.44), while the same association was significant at higher values of hepcidin (OR = 2.84; 95% CI 1.32–6.09) (Table 4). This joint effect is reflected in the adjusted probabilities for iron deficiency; higher predicted probabilities were found for children with *H. pylori* infection and higher levels of hepcidin (0.24 CI 95% 0.14–0.34), and for children without *H. pylori* infection and lower levels of hepcidin (0.24 CI 95% 0.14–0.33) (Figure 1). 

Age also was associated with iron deficiency: for each year of increase in age, the children had a decrease in the odds of presenting iron deficiency by a factor of −0.22 (β coefficient), OR = 0.80 (CI95% 0.68–0.95, *p* = 0.012). Schoolchildren with stunting or risk of stunting had an odds 2.39 times larger of presenting iron deficiency than those with normal height to age, holding the other variables in the model constant (Table 4). 

## 4. Discussion

In this study, we found that the association between *H. pylori* and iron deficiency is different by hepcidin levels: *H. pylori* infection was associated with iron deficiency in children with higher levels of hepcidin; but this association was not statistically significant in children with lower hepcidin levels. These results suggest that in children with *H. pylori* infection and iron deficiency, the hepcidin synthesis is upregulated. 

Iron deficiency, anemia, *H. pylori* infection, and chronic inflammation are common causes of morbidity in children, and they often occur simultaneously, particularly in low- and middle-income countries. Some studies have found an association between *H. pylori* infection and iron deficiency or with iron deficiency anemia in children and adolescents [30,31]. The mechanisms that explain this association still have not been clarified. Hepcidin plays an important role in the iron metabolism; its synthesis decreases in iron deficiency and iron deficiency anemia and increases by inflammatory stimulus [3]. Specifically in *H. pylori* infection, a chronic infection that involves an inflammatory response, the increases in the hepcidin synthesis can reduce the amount of iron potentially available to infecting microorganisms [32], but it can also contribute to iron deficiency and anemia because high hepcidin levels lead to reduced iron absorption and its recirculation. The relationship between infection and iron deficiency is bidirectional and responds to different mechanisms—not all of them clearly understood.

Only two studies with controversial results have been reported on the relationship between *H. pylori* infection and iron deficiency based on hepcidin levels in children. Korean children with iron deficiency anemia who received iron supplementation showed improvement in their iron status. However, the group of children with *H. pylori* infection had a lower response to iron therapy than the group without the infection; in addition, the children with *H. pylori* infection had higher hepcidin levels at baseline and 3 months after of iron supplementation compared to the children with iron deficiency anemia without the *H. pylori* infection. The results are similar to ours. We also found higher hepcidin concentration in children with *H. pylori* infection. In that study, *H. pylori* infection was diagnosed using monoclonal fecal antigen immunoassay techniques that are useful in detecting current infection [33]; in our study, urea breath test was used to determinate active *H. pylori* infection. In the other study, *H. pylori* bacterium was detected by IgG antibodies to *H. pylori*; the hepcidin levels were not different among the *H. pylori* seropositive or seronegative children. In addition, the *H. pylori* seropositive children with low hepcidin levels were more likely to have iron deficiency than the seropositive children with high hepcidin levels. These results imply that not all children with infection show an increased serum hepcidin level, thus the association between in *H. pylori* infection and iron deficiency might be explained by other mechanisms than hepcidin production [34]. However, the serologic method used to detect *H. pylori* also can imply that some children may have had a prior inactive infection by *H. pylori* [35].

In looking at hepcidin concentration and iron status in response to treatment for *H. pylori* in an adult population with *H. pylori* infection and iron deficiency anemia, Sapmaz et al. measured serum hepcidin levels and iron status indicators before and after eradication treatment of *H. pylori*. Pre-treatment hepcidin levels were high and iron markers were low, and there was a significant improvement in iron parameters as well as a decrease in the concentration of hepcidin in patients with successful eradication compared with patients in whom eradication treatment was not successful. The authors concluded that *H. pylori* infection increases the concentration of hepcidin, and, when the bacterium is eradicated, hepcidin concentration decreases and indicators of iron status—including serum iron, ferritin, hemoglobin, mean cell volume, total iron binding capacity, and transferrin—improve [36].

In others studies, the relation between hepcidin levels and iron deficiency has been evaluated in other infections. In patients with malaria or HIV infection, a reduction in the production of hepcidin in anemic individuals has been observed, despite the stimulus of the infectious condition [37]. It is important to note that in our study, no children had anemia—they had low iron stores, a state commonly used to identify iron deficiency, which is a phase that can tend to iron deficiency anemia. Thus, a prolonged infection or a chronic inflammatory status will lead to iron deficiency and anemia due to the blockage of iron absorption and recirculation in response to high hepcidin levels [32]. However, iron deficiency anemia or a low iron status should trigger lower hepcidin production to increase iron absorption, on the one hand, with the potential undesirable effect of increasing the virulence of the infecting pathogen but enhancing the body’s own immune response, on the other hand. Neither the timing of these events nor what drives hepcidin increased/decreased secretion in the presence of infection, inflammation, and iron deficiency is completely understood [38].

In our study, although we were limited by the cross-sectional design that precludes making causal inferences or establishing the sequence of events, we found that, in the presence of infection with *H. pylori*, there was higher hepcidin levels, as would be expected. Likewise, we found that hepcidin levels were lower when iron stores were low, but this was statistically significant only when children were free of *H. pylori* infection. In contrast to what was found when the children had both conditions, *H. pylori* infection and iron deficiency, in these cases the hepcidin levels were higher. These results suggest that in children with *H. pylori* infection, the hepcidin synthesis is upregulated regardless of their iron status. Therefore, the stimulus to the synthesis of hepcidin due to *H. pylori* infection is greater than iron deficiency stimulus; in children with iron deficiency who have *H. pylori* infection, hepcidin levels are higher than those in children with iron deficiency without *H. pylori* infection. If the infection is prolonged, as is common in *H. pylori* infection, or the individual is malnourished, the hypoferremia caused by inflammation may produce or worsen the nutritional deficiency [25]. These results could indicate that in children with iron deficiency or iron deficiency anemia and *H. pylori* infection, it is necessary to treat the infection first and then determine whether it is still necessary to give iron supplements. Future studies are needed to understand the hierarchical nature of hepcidin production, including production under normal and pathological conditions, also to identify cut-off values that classify low, normal, or high hepcidin status. Likewise, more research is needed to explore if other chronic inflammatory processes besides *H. pylori* infection are associated with iron deficiency and high production of hepcidin. 

In relation to acute-phase proteins, it was interesting to note that under univariate analysis the levels of hepcidin were different in groups with and without iron deficiency. Children with high levels of CRP had higher hepcidin concentration, but this difference was statistically significant only in those without iron deficiency. In contrast to what was found with AGP, hepcidin concentration was higher in children with high AGP, but the difference was statistically significant only in children with iron deficiency. However, in multivariate analysis, the association between these acute-phase proteins with iron deficiency was not statistically significant. It should be noted that these acute-phase proteins have different behavior during the infection–inflammation process. CRP returns to normal faster than AGP, which is considered a late marker of inflammation and may remain high even in established chronic processes. In addition, CRP and ferritin show similar behavior in response to infection—rapid increase with onset of inflammation—however, ferritin remains elevated longer than CRP [25]. For this reason, a possible misclassification of ‘iron deficiency’ can be present in this study, as ferritin concentration may go up as part of the acute phase response to infectious/inflammatory processes. As part of our analysis, we applied a correction factor to ferritin values, as suggested by Thurnham D et al., including 0.67 for elevated CRP and 0.73 for elevated AGP [39]. The frequency of iron deficiency with and without this adjust was similar (18.7%, *n* = 62 vs. 16.0%, *n* = 56), and the results were essentially the same for both simple and multivariate analyses. Given that there is no general consensus in the literature about the use of these correction factors, we decided not to use them. Also, as had been mentioned, some authors recommend excluding from the analysis cases with high values of acute phase proteins; however, in the context of our study, this is not particularly helpful because the type of conditions we were studying, which include infections and nutritional deficiencies (particularly iron deficiency), often occur simultaneously in children.

Several limitations of our study need to be acknowledged. As has been noted previously, this was a cross-sectional design, so we cannot derive directional or causal inferences from our results. Likewise, we could not document the sequence of events between infection with *H. pylori* and hepcidin response nor the behavior of inflammatory markers and the associated hepcidin response, and the onset of iron deficiency. Other limitation is that it was unknown if some children participating in this study had received iron supplementation in the previous year, although we do know that at least in the 6 months previous to blood sampling in this study, they had not received iron supplements.

Despite these gaps in our understanding of hepcidin production in the presence of chronic infection and inflammation, our study adds to our understanding of this relationship and points out further avenues of investigation that will better inform clinical management of iron deficiency and anemia in children with *H. pylori* infection. 

## 5. Conclusions

The association between *H. pylori* and iron deficiency differs by hepcidina levels; the probabilities of having iron deficiency are higher for children with *H. pylori* infection and higher levels of hepcidin, and for children without *H. pylori* infection and lower levels of hepcidin. These results suggest two possibilities related to the usefulness of hepcidin determination in children: (1) Children without *H. pylori* infection with low hepcidin concentration may have higher probabilities of presenting iron deficiency. (2) Children with *H. pylori* infection and higher hepcidin values may also have higher probability of iron deficiency. The difference is that, in the first case, children may benefit from receiving iron supplementation, while in the second case, before offering iron supplementation, it may be important to offer eradication treatment for the underlying infection and then evaluate iron status to assess if treatment is still needed. In the latter scenario, it is possible that the reason for low iron status is not a low intake but rather a downregulation of iron absorption. If this is the case, providing additional iron via oral supplementation will cause an iron overload, which may release reactive oxygen species, further damaging the intestinal mucosa.

## Figures and Tables

**Table 1:** Characteristics of the study population by iron nutritional status.

Variables	Iron Deficiency (Ferritin < 15 ng/mL) *n* = 56		Normal (Ferritin ≥ 15 ng/mL) *n* = 294		*p* Value
	*n*	%	*n*	%	
**Age (years) ^a^**	9.1 ± 1.8		9.8 ± 1.8		0.009
**Sex**					
Male	25	44.6	132	44.9	
Female	31	55.4	162	55.1	0.972
**Body mass index (Z score) ^a^**	0.36 ± 0.90		0.65 ± 1.11		0.069
**Body mass index ^b^**					
Normal	43	76.8	188	64.0	
Overweight/Obese	13	23.2	106	36.0	0.063
**Height for age (Z score) ^a^**	−0.66 ± 1.11		−0.32 ± 0.97		0.020
**Height for age ^c^**					
Normal	32	57.1	234	79.6	
Risk of stunting/Stunting	24	42.9	60	20.4	0.000
***H. pylori* infection**					
Negative	36	64.3	237	80.6	
Positive	20	35.7	57	19.4	0.007
**Hemoglobin (g/dL) ^a^**	14.18 ± 0.84		14.22 ± 0.83		0.793
**Hepcidin (ng/mL) ^d^**	6.9 (3.5–14.1)		8.6 (4.2–15.4)		0.261
**C-reactive protein ^d^**	0.00 (0.00–0.54)		0.00 (0.00–1.52)		0.069
Normal < 5 mg/L	52	92.9	257	88.0	
High ≥ 5 mg/L	4	7.1	35	12.0	0.293
**α1-glycoprotein ^d^**	0.65 (0.50–0.84)		0.60 (0.47–0.80)		0.160
Normal < 1 g/L	47	83.9	262	89.1	
High ≥ 1 g/L	9	16.1	32	10.9	0.269

^a^ Mean ± SD; ^b^ BMI categories: Normal = 1 SD > *z* score > −2 SD, Overweight/Obese = *z* score ≥ 1 SD; ^c^ Height for age: Normal = *z* score ≥ −1 SD, Risk of stunting/Stunting = *z* score < −1 SD; ^d^ Median (p25–p75).

**Table 2:** Serum hepcidina levels in children by iron nutritional status.

Variables		Hepcidin Level (ng/mL) ^a^			*p* Value
	*n*	Iron Deficiency (Ferritin < 15 ng/mL) (*n* = 56)	*n*	Normal (Ferritin ≥ 15 ng/mL) (*n* = 294)	
**Height for age ^b^**					
Normal	32	6.1 (2.9–12.0)	234	8.7 (4.3–15.8)	0.045
Risk to stunting/Stunting	24	7.7 (5.4–18.8)	60	8.4 (4.0–12.9)	0.500
*p* value		0.079		0.513	
**Body mass index ^c^**					
Normal	43	8.2 (4.7–17.7)	188	9.0 (4.1–15.8)	0.965
Overweight/Obesity	13	3.6 (0.3–6.4)	106	8.0 (4.7–14.0)	0.012
*p* value		0.019		0.807	
***H. pylori* infection**					
Negative	36	5.5 (2.4–10.4)	237	8.2 (4.2–14.0)	0.017
Positive	20	10.5 (6.4–20.1)	57	10.4 (5.9–19.2)	0.693
*p* value		0.094		0.105	
**C-reactive protein**					
Normal (<5 mg/L)	52	7.0 (3.1–15.6)	257	8.4 (4.2–14.0)	0.456
High (≥5 mg/L)	4	6.7 (5.7–8.7)	35	15.0 (4.4–32.3)	0.287
*p* value		0.924		0.016	
**α1-glycoprotein**					
Normal (<1 g/L)	47	6.8 (2.8–13.9)	262	8.3 (4.2–15.1)	0.090
High (≥1 g/L)	9	10.5 (6.6–20.9)	32	12.0 (4.7–18.1)	0.729
*p* value		0.044		0.132	

^a^ Median (p25–p75); ^b^ BMI categories: Normal = 1 SD > *z* score > −2 SD, Overweight/Obese = *z* score ≥1 SD; ^c^ Height for age: Normal = *z* score ≥ −1 SD, Risk of stunting/Stunting = *z* score < −1 SD.

**Table 3:** Iron status, hepcidin, and inflammation markers by *H. pylori* infection.

Biomarkers	*H. pylori* (+) *n* = 77		*H. pylori* (−) *n* = 273		*p* Value
	*n*	%	*n*	%	
**Hemoglobin (g/dL) ^a^**	14.0 ± 0.8		14.2 ± 0.8		0.017
**Ferritin (ng/mL) ^b^**	26.5 (13.6–41.4)		31.3 (21.2–43.3)		0.110
Normal ≥ 15 ng/mL	57	74.0	237	86.8	
Low levels < 15 ng/mL	20	26.0	36	13.2	0.007
**Hepcidin (ng/mL) ^b^**	10.4 (6.0–19.3)		7.5 (4.0–13.8)		0.005
**C-reactive protein**					
Normal < 5 mg/L	69	90.8	240	88.2	
High ≥ 5 mg/L	7	9.2	32	11.8	0.533
**α1-glycoprotein**					
Normal < 1 g/L	58	75.3	251	91.9	
High ≥ 1 gL	19	24.7	22	8.1	0.000

^a^ Arithmetic mean ± SD; ^b^ Median (p25–p75).

**Table 4:** Association between *H. pylori* infection and iron deficiency by hepcidin levels. Multivariate analysis.

Variable	Odds Ratio ^a^	CI 95%	*p* Value
**Age (years)**	0.80	0.68–0.95	0.012
**Height for age (*Z* score) ^b^**			
Normal	1.00		
Stunting/risk to stunting	2.39	1.26–4.52	0.008
**With *H. pylori* infection by hepcidin levels ^c^**			
First quartile	0.17	0.02–1.44	0.104
Second–Fourth quartiles	2.84	1.32–6.09	0.007
**C-Reactive protein**			
Normal < 5 mg/L	1.00		
High ≥ 5 mg/L	0.44	0.12–1.53	0.195
**α-1 Glycoprotein**			
Normal < 1 g/L	1.00		
High ≥ 1 g/L	1.61	0.60–4.33	0.348

Pearson *χ*^2^ goodness-of-fit test 348.41, *p* value 0.309. ^a^ Adjusted odd ratio logistic model. Adjusted also by sex and body mass index (overweight/obesity vs. normal BMI); ^b^ Height for age: Normal = *z* score ≥ −1 SD, Risk of stunting/Stunting = *z* score < −1 SD; ^c^ Hepcidin categories: first quartile with values between 0.00–4.18 ng/mL and second to fourth quartiles with values between 4.19–89.93 ng/mL.
